# Cadherin-11 Regulates Macrophage Development and Function

**DOI:** 10.3389/fimmu.2022.795337

**Published:** 2022-02-08

**Authors:** Sarah To, Thandiwe Chavula, Mesias Pedroza, Jennifer Smith, Sandeep K. Agarwal

**Affiliations:** Department of Medicine, Section of Immunology, Allergy and Rheumatology, Baylor College of Medicine, Houston, TX, United States

**Keywords:** Cadherin-11, macrophages, monocytes, pulmonary fibrosis, polarization, phagocytosis

## Abstract

Cadherin-11 (CDH11) is a cell-cell adhesion protein that has previously been reported to play an important role in the pathogenesis of pulmonary fibrosis. It is expressed on macrophages in the fibrotic lung. However, the role of CDH11 on macrophage biology has not yet been studied. We show using immunophenotypic analyses that *Cdh11^-/-^* mice have fewer recruited monocyte-derived macrophages and Ly6C^hi^ monocytes in the lungs compared to wild-type mice in the intraperitoneal bleomycin-induced pulmonary fibrosis model. Additionally, fewer Ly6C^hi^ monocytes were detected in the bone marrow and peripheral blood of naive *Cdh11^-/-^* mice. Given that macrophages are derived from monocytes, we investigated the precursors of the monocyte/macrophage lineage in the bone marrow. We found increased numbers of CMPs and reduced numbers of GMPs and MPs/cMoPs in *Cdh11^-/-^* mice compared to wild-type mice, suggesting decreased differentiation towards the myeloid lineage in *Cdh11^-/-^* mice. Furthermore, we show using bone marrow cells that loss of CDH11 impaired monocyte to macrophage differentiation. We also demonstrate that CDH11 deficiency repressed the M2 program and impaired the phagocytic function of bone marrow-derived macrophages. Overall, our findings demonstrate a role for CDH11 in macrophage development, M2 polarization, and phagocytic function.

## Introduction

Pulmonary fibrosis is a chronic lung disease characterized by excessive deposition of extracellular matrix (ECM) proteins by myofibroblasts leading to remodeling of the lung architecture (1). Despite extensive research, the mechanisms underlying the pathogenesis of lung fibrosis remain incompletely understood. Current therapeutic strategies for the treatment of pulmonary fibrosis are not curative and limited to delaying disease progression (2). Thus, there is a need to understand the molecular and cellular mechanisms involved in lung fibrosis in order to identify targets for intervention to stop the disease from progressing.

Macrophages are the key players in lung fibrosis. Under normal homeostatic conditions, two main populations of macrophages are found in the lung: tissue-resident alveolar macrophages (TrAMs), which line the inner alveolar surface, and interstitial macrophages (IMs), which reside in the lung interstitium. During lung injury, monocytes infiltrate the lungs from the bone marrow and differentiate into a second population of alveolar macrophages (monocyte-derived alveolar macrophages [MoAM]) that is ontogenically distinct from tissue-resident alveolar macrophages (3–6). Several studies have shown a critical role for MoAMs in the development of lung fibrosis (5–8).

Macrophages are highly plastic cells and can polarize into specific functional phenotypes depending on the local environmental cues (9). They can polarize to classically activated (M1) macrophages in response to LPS and IFNγ or alternatively activated (M2) macrophages in response to IL-4 and IL-13 (10, 11). At steady state, lung macrophages have been found to express both M1 and M2 markers (5, 12). However, during lung fibrosis, several studies have shown a predominant infiltration of macrophages biased towards an M2 phenotype into the lung (13–18). Activated M2 macrophages produce several profibrotic mediators such as transforming growth factor β (TGF-β), platelet-derived growth factor (PDGF), and CCL18 that induce fibroblast activation and promote myofibroblast proliferation (8, 13, 17, 19–22).

Cadherin-11 (CDH11) is a cell-cell adhesion protein that has been reported to play an important role in the pathogenesis of fibrosis of the lungs, skin, liver, heart, and intestines (23–28). Our group has previously shown that CDH11 expression is increased in the lungs of patients with idiopathic pulmonary fibrosis and in mice given bleomycin (23). Furthermore, we showed that mice deficient in CDH11 or inhibition of CDH11 with neutralizing monoclonal antibodies against CDH11 markedly protected the mice from bleomycin-induced lung fibrosis (23). In the fibrotic lung, CDH11 is expressed on fibroblasts, injured type II alveolar epithelial cells, as well as alveolar macrophages (23). We previously showed that CDH11 may be promoting lung fibrosis by regulating the production of TGF-β1 in alveolar macrophages and epithelial to mesenchymal transition (EMT) in alveolar epithelial cells (23). More recently, Lodyga and colleagues showed that CDH11 can also mediate the adhesion of macrophages to myofibroblasts which maintains the TGF-β1-producing macrophages in close proximity for persistent activation of the profibrotic myofibroblasts to promote the development of lung fibrosis (29).

The role of CDH11 on macrophage biology has not yet been studied. In the present study, we immunophenotype the lungs of wild type and *Cdh11*-deficient (*Cdh11^-/-^*) mice challenged with intraperitoneal bleomycin and found reduced numbers of Ly6C^hi^ monocytes and recruited monocyte-derived macrophages in *Cdh11^-/-^* mice. Naive *Cdh11^-/-^* mice also have fewer Ly6C^hi^ monocytes in the bone marrow and peripheral blood. Furthermore, we show that CDH11 regulates the development of macrophages and alters its M2 program and phagocytic function. Collectively, our results provide insight into how CDH11 regulates macrophage biology during pulmonary fibrosis and suggests that targeting CDH11 on macrophages could be a viable therapeutic strategy for the prevention and treatment of pulmonary fibrosis.

## Materials and Methods

### Mice

Cadherin-11 deficient (*Cdh11^-/-^*) and wild type (WT) mice on a mixed C57BL/6 and C129 background (23, 30, 31) were housed at Baylor College of Medicine in accordance with institutional and NIH guidelines. All studies using these mice were conducted with approval by the Institutional Animal Care and Use Committee (IACUC) of Baylor College of Medicine.

### Bleomycin-Induced Pulmonary Fibrosis

Male mice (4-6 week old) were injected intraperitoneally with bleomycin (TEVA Pharmaceuticals) at 0.035 U/g twice weekly on days 1, 4, 8, 11, 15, and 18 for 3 weeks to induce pulmonary fibrosis (32). Control mice received intraperitoneal PBS. Lungs were harvested on day 21 (3 days after the 6th injection).

### Cell Isolation

Bone marrow cells were collected by flushing both femurs and tibias from 8-12 week old male WT and *Cdh11^-/-^* mice with ice-cold PBS containing 0.2% FBS and filtered through a 40 µm cell strainer. Lung cells were collected by mechanical disruption and digested with 1 mg/mL Collagenase Type 4 (Worthington) and 0.1 mg/mL DNase I (Worthington). The lung digest was filtered through a 100 µm cell strainer. Primary alveolar macrophages were isolated from bronchoalveolar lavage fluid (BALF) collected by tracheostomy. Whole blood was collected from 8-10 week old male WT and *Cdh11^-/-^* mice by cardiac puncture into EDTA-coated collection tubes.

### Bone Marrow-Derived Macrophage Culture

For analysis of bone marrow (BM) differentiation, BM cells were seeded onto bacterial Petri dishes and cultured in DMEM supplemented with 20% FBS and 20 ng/mL recombinant murine M-CSF (PeproTech) for 1, 3, 5, or 7 days. The medium was replenished on day 4. Non-adherent cells were removed, and adherent cells were harvested at the indicated time points for flow analysis. To generate bone marrow-derived macrophages (BMDMs) for M1/M2 polarization and phagocytosis studies, BM cells were cultured with 50 ng/mL M-CSF for 7 days. The medium was replenished on day 4. Adherent cells were harvested on day 7 and used as BMDMs. To induce M1 macrophages, BMDMs were primed with IFNγ (50 ng/mL, R & D Systems) for 6 hrs followed by stimulation with LPS (100 ng/mL, R & D Systems) for 18 hrs. For analysis of *Nos2* mRNA expression, BMDMs were primed with IFNγ for 18 hrs and stimulated with LPS for 2 hrs. To induce M2 macrophages, BMDMs were stimulated with IL-4 (20 ng/mL, R & D Systems) for 24 hrs. For analysis of *Retnla* mRNA expression, BMDMs were stimulated with IL-4 for 4 hrs. For analysis of TGF-β1 secretion in culture supernatants, BMDMs were stimulated with IL-4 for 72 hrs.

### Flow Cytometry

Macrophage formation from BM was assessed by staining cells with CD11b-BUV395 (clone M1/70; BD Biosciences) and F4/80-FITC (clone BM8; BioLegend) at 4°C for 30 minutes. M1 and M2 markers were analyzed on BMDMs using CD86-AF700 (clone GL1; BD Biosciences), CD206-PerCP-Cy5.5 (clone C086C2; BioLegend) and CD80-PE-CF594 (clone 16-10A1; BD Biosciences).

For analysis of monocytes and macrophages in the lungs, single lung cell suspensions were stained with the following fluorochrome-conjugated antibodies at 4°C for 30 minutes: CD45-APC-Cy7 (clone 30F11; BD Biosciences), Ly6G-BV510 (clone 1A8; BioLegend), F4/80-PE-CF594 (clone T45-2342; BD Biosciences), CD24-BV786 (clone M1/69; BD Biosciences), CD64-BV650 (clone X54-5/7.1; BD Biosciences), CD11c-APC (clone HL3; BD Biosciences), CD11b-BUV395 (clone M1/70; BD Biosciences), Siglec-F-APC-R700 (clone E50-2440; BD Biosciences), MHCII-PE-Cy5 (clone M5/114.15.2; BioLegend), Ly6C-FITC (clone AL-21; BD Biosciences), CD204-PE (clone REA148; Miltenyi Biotec), and CD206-PE-Cy7 (clone C068C2; BioLegend).

For peripheral blood analysis, 100 µL of whole blood was stained with the following fluorochrome-conjugated antibodies at 4°C for 30 minutes: CD45-APC-Cy7 (clone 30F11; BD Biosciences), Ly6G-FITC (clone 1A8; BD Biosciences), CD11b-PE-Cy5 (clone M1/70; BioLegend), Ly6C-PE-Cy7 (clone HK1.4; BioLegend) and CD115-BV605 (clone AFS98; BioLegend).

For analysis of bone marrow monocytes and neutrophils, BM cells were stained with the following fluorochrome-conjugated antibodies at 4°C for 30 minutes: CD135-PE (clone A2F10.1; BD Biosciences), CD115-BV421 (clone AFS98; BioLegend), c-Kit-FITC (CD117, clone 2B8; BD Biosciences), CD11b-PE-Cy5 (clone M1/70; BioLegend), Ly6C-PE-Cy7 (clone AL-21; BD Biosciences), Ly6G-APC-Cy7 (clone 1A8; BD Biosciences) and a lineage cocktail containing CD3e-APC (clone 145-2C11; BD Biosciences), CD19-APC (clone 1D3; BD Biosciences), and NK-1.1-APC (clone PK136; BD Biosciences). For analysis of hematopoietic stem cells (HSCs) and myeloid progenitor cells, BM cells were first incubated with CD16/CD32-PerCP-Cy5.5 (clone 2.4G2; BD Biosciences) at 4°C for 10 minutes followed by incubation with the following fluorochrome-conjugated antibodies at 4°C for 30 minutes (except for CD34 which was incubated for 70 minutes): CD34-PE (clone RAM34; BD Biosciences), CD135-BV421 (clone A2F10; BioLegend), CD115-BV605 (clone AFS98; BioLegend), c-Kit-FITC (CD117, clone 2B8; BD Biosciences), Sca1-PE-Cy7 (Ly-6A/E, clone D7; BD Biosciences), Ly6C-APC-Cy7 (clone HK1.4; BioLegend) and a lineage cocktail (BD Biosciences) supplemented with CD4-APC (clone RM4-5; BD Biosciences) and CD8a-APC (clone 53-6.7; BD Biosciences). Cells were stained with Live/Dead Fixable blue stain (Molecular Probes) for exclusion of dead cells. All data were acquired on the BD LSRII at the Flow Cytometry Core Facility at Baylor College of Medicine (Houston, Texas) using the BD FACSDiva Software (BD Biosciences). Compensation and analyses were performed using the FlowJo v10.7 Software (BD Life Sciences).

### Quantitative Real-Time PCR

For analysis of mRNA expression in cultured cells, cDNA was generated using the Cell-to-CT kit (Ambion) according to the manufacturer’s protocol. Quantitative real-time PCR was performed on an Applied Biosystems Step One Plus PCR System (Applied Biosystems) using TaqMan Gene Expression Assays (ThermoFisher Scientific) for mouse *Arg1* (Mm00475988_m1), *Chil3* (Mm00657889_mH), *Retnla* (Mm00445109_m1), *Msr1* (Mm00446214_m1), *Mrc1* (Mm01329362), *Nos2* (Mm00440502_m1), and *Rn18s* (Mm03928990_g1). Transcript levels were normalized to *Rn18s*, and relative expression was calculated using the comparative Ct method (ΔΔCt).

### ELISA

Cytokine concentrations in the culture supernatants were determined using the Mouse TGF-beta 1 DuoSet ELISA kit (R&D Systems) or the Mouse TNF alpha, IL-6, or IL-12 ELISA Kits (Invitrogen). Values were quantified against a standard curve.

### Phagocytosis Assay

For analysis of phagocytosis by flow cytometry, BMDMs or primary alveolar macrophages were incubated with pHrodo-green conjugated Zymosan bioparticles (Molecular Probes) at 50 µg bioparticles/100,000 cells. Following synchronization by centrifugation, the cells were allowed to internalize the bioparticles for 30 to 120 minutes at 37°C. Non-internalized particles were removed by washing with ice cold PBS. The cells were fixed and analyzed by flow cytometry. Phagocytosis was identified as pHrodo-green-positive cells. Cells incubated with bioparticles on ice were used as negative controls.

For analysis of phagocytosis by immunofluorescence, BMDMs were seeded into chamber slides and exposed to Alexa Fluor 594-conjugated Zymosan bioparticles (Invitrogen) at a ratio of 1:10 (cells:bioparticles) and incubated at 37°C for 2 hrs. Uningested particles were removed by washing with PBS. The cells were fixed, permeabilized, and stained for F-actin with Alexa Fluor 488-conjugated phalloidin. The slides were then mounted in ProLong Antifade with DAPI (Invitrogen). Ingested particles were counted under a microscope at 40x magnification.

### Statistical Analysis

All statistical analyses were performed using GraphPad Prism v6 Software. Prior to statistical significance analysis, all data were first tested for normality using the D’Agostino-Pearson or Shapiro-Wilk test. For normally distributed data, the two-tailed unpaired parametric Student’s *t*-test was used to compare the means of two groups. The one-way ANOVA with Bonferroni *post-hoc* test was used for multiple comparisons. When the data were not normally distributed, we log2 transformed the data. If the log2 transformed data was normally distributed, we performed statistical analysis on the log2-transformed data. If the log2 transformed data was not normally distributed, we performed the non-parametric Mann Whitney U test on the non-transformed data. All data are presented as mean ± SEM. Numbers of mice and samples analyzed are indicated in the figure legends. A *P* value of <0.05 was considered statistically significant and indicated with asterisks (* P<0.05; ** P<0.01; *** P<0.001; **** P<0.0001).

## Results

### *Cdh11^-/-^* Mice Have Fewer Monocyte-Derived Macrophages and Fewer Ly6C^hi^ Monocytes in the Lung Compared to WT Mice After Bleomycin Administration

**Figure 1:**
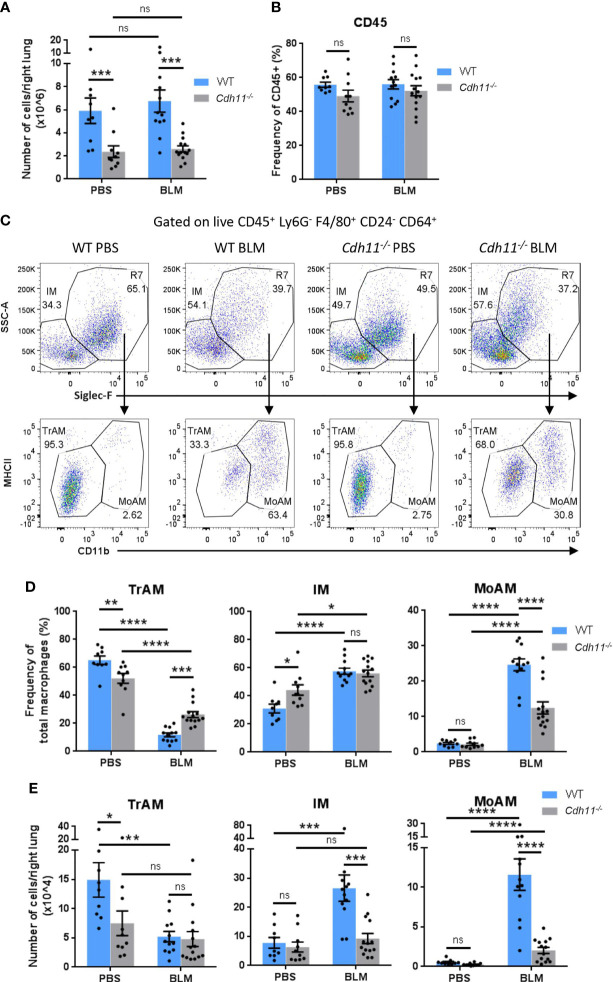
*Cdh11^-/-^* mice have fewer monocyte-derived macrophages in the bleomycin-induced pulmonary fibrosis model. **(A)** Total number of cells per right lung and **(B)** frequency of CD45^+^ cells in lungs of *Cdh11^-/-^* or wild type (WT) mice 21 days after IP bleomycin (BLM) administration. PBS administered mice were used as the control. **(C)** Representative flow cytometry plots showing the relative abundance of each macrophage subset in the mouse lung for each experimental group. **(D)** Frequencies of TrAMs, IMs, and MoAMs as a percentage of total macrophages. **(E)** Total number of TrAMs, IMs, and MoAMs per right lung. Total numbers of each macrophage subset were calculated by multiplying the frequency of live (determined from flow cytometry) by the total number of lung cells (obtained using a hemocytometer). Data are pooled from four independent experiments and represent mean ± SEM, n=9 (WT PBS), n=12 (WT BLM), n=10 (*Cdh11^-/-^* PBS), n=14 (*Cdh11^-/-^* BLM). Statistical significance was assessed using one-way ANOVA with Bonferroni *post-hoc* test. *P<0.05, **P<0.01, ***P<0.001, ****P<0.0001, ns, non-significant.

CDH11 is expressed on alveolar macrophages in the fibrotic lung (23, 29). However, the role of CDH11 in macrophage biology has not yet been studied. Here, we use the intraperitoneal (IP) bleomycin model of pulmonary fibrosis to characterize the macrophage subsets, as well as their monocyte precursors, in the lung tissue of WT and *Cdh11^-/-^* mice. We evaluated the cell subsets after 21 days bleomycin administration, when substantial lung fibrosis is detected (**Supplementary Figure 1**). Flow cytometry was performed, and the gating strategy used to identify lung macrophages and monocytes is outlined in **Supplementary Figure 2**. The total number of lung cells from PBS-treated WT mice was similar to that from bleomycin-treated WT mice (**Figure 1A**). However, PBS and bleomycin-treated *Cdh11^-/-^* mice had significantly fewer numbers of total lung cells compared to WT mice. The frequency of CD45^+^ cells was not affected by the genotype of the mice or bleomycin administration (**Figure 1B**). Analysis of macrophage subsets showed that *Cdh11^-/-^* mice had a reduced frequency and fewer number of tissue-resident alveolar macrophages (TrAMs) (identified as CD45^+^ Ly6G^-^ F4/80^+^ CD64^+^ CD24^-^ Siglec-F^+^ SSC^hi^ CD11b^-^) compared to WT mice (**Figures 1C–E**). Following bleomycin administration, both the frequency and number of TrAMs were reduced in WT mice. In contrast, the frequency of TrAMs in *Cdh11^-/-^* mice was reduced to a lesser extent compared to WT mice after bleomycin administration while the total number of TrAMs was not affected. Interestingly, interstitial macrophages (IMs) (identified as CD45^+^ Ly6G^-^ F4/80^+^ CD64^+^ CD24^-^ Siglec-F^-^ SSC^low^) and monocyte-derived alveolar macrophages (MoAMs) (identified as CD45^+^ Ly6G^-^ F4/80^+^ CD64^+^ CD24^-^ Siglec-F^+^ SSC^hi^ CD11b^+^) increased in both frequency and total number after bleomycin administration in WT mice and the number of both populations were markedly reduced in *Cdh11^-/-^* mice. These data suggest that the fewer numbers of IMs and MoAMs in *Cdh11^-/-^* mice may contribute to the attenuation of pulmonary fibrosis in these mice in the IP bleomycin model.

**Figure 2:**
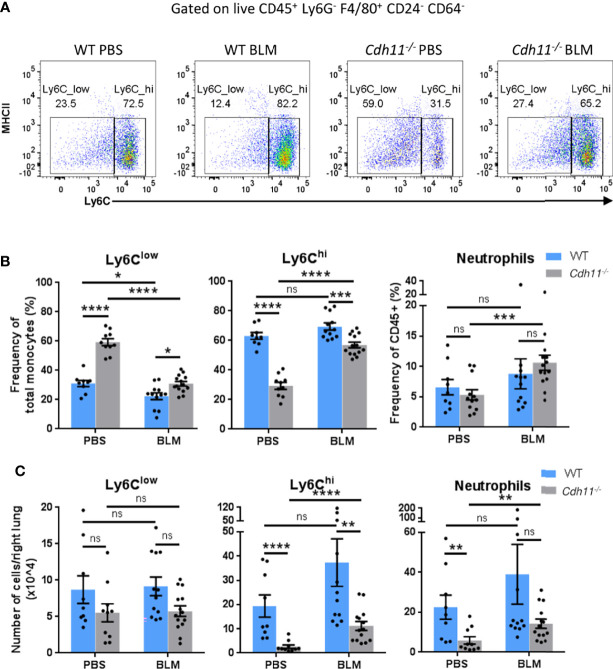
*Cdh11^-/-^* mice have fewer Ly6C^hi^ monocytes in the lung. **(A)** Representative flow cytometry plots showing the relative abundance of Ly6C^low^ and Ly6C^hi^ monocytes in the lungs of *Cdh11^-/-^* or WT mice 21 days after bleomycin (BLM) administration. PBS administered mice were used as the control. **(B)** Frequency of Ly6C^low^ and Ly6C^hi^ monocytes as a percentage of total monocytes and frequency of neutrophils as a percentage of total CD45^+^ cells. **(C)** Total number of Ly6C^low^ and Ly6C^hi^ monocytes and neutrophils per right lung. Total numbers of each cell population were calculated by multiplying the frequency of live (determined from flow cytometry) by the total number of lung cells (obtained using a hemocytometer). Data are pooled from four independent experiments and represent mean ± SEM, n=9 (WT PBS), n=12 (WT BLM), n=10 (*Cdh11^-/-^* PBS), n=14 (*Cdh11^-/-^* BLM). Statistical significance was assessed using one-way ANOVA with Bonferroni *post-hoc* test. * P<0.05, **P<0.01, ***P<0.001, ****P<0.0001, ns, non-significant.

Given that recruited inflammatory macrophages originate from bone marrow monocytes, we analyzed the monocyte populations in WT and *Cdh11^-/-^* mice before and after bleomycin administration. PBS and bleomycin-treated *Cdh11^-/-^* mice had a higher frequency of Ly6C^low^ monocytes (identified as CD45^+^ Ly6G^-^ F4/80^+^ CD64^-^ CD24^-^ MHCII^-^ Ly6C^low^) compared to WT mice (**Figures 2A, B**). However, the frequency of Ly6C^low^ monocytes was reduced to a larger extent in *Cdh11^-/-^* mice compared to WT mice after bleomycin administration. The total number of Ly6C^low^ monocytes was similar between *Cdh11^-/-^* and WT mice before and after bleomycin administration (**Figure 2C**). In contrast, PBS-treated *Cdh11^-/-^* mice had a significantly lower frequency and total number of Ly6C^hi^ monocytes compared to WT mice. No significant difference in the frequency or total number of Ly6C^hi^ monocytes was observed in WT mice after bleomycin administration. However, both the frequency and total number of Ly6C^hi^ monocytes increased in *Cdh11^-/-^* mice after bleomycin administration but were significantly lower compared to WT mice. No remarkable differences in neutrophils or other leukocyte subsets were observed between WT and *Cdh11^-/-^* mice treated with bleomycin (**Figures 2B, C**; **Supplementary Figures 3**, **4**). Collectively, these data suggest that the fewer numbers of recruited monocyte-derived macrophages in the bleomycin-treated *Cdh11^-/-^* mice may be due to fewer numbers of Ly6C^hi^ monocyte precursors.

### *Cdh11^-/-^* Mice Have Fewer Ly6C^hi^ Monocytes and Monocyte Progenitors in the Bone Marrow

**Figure 3:**
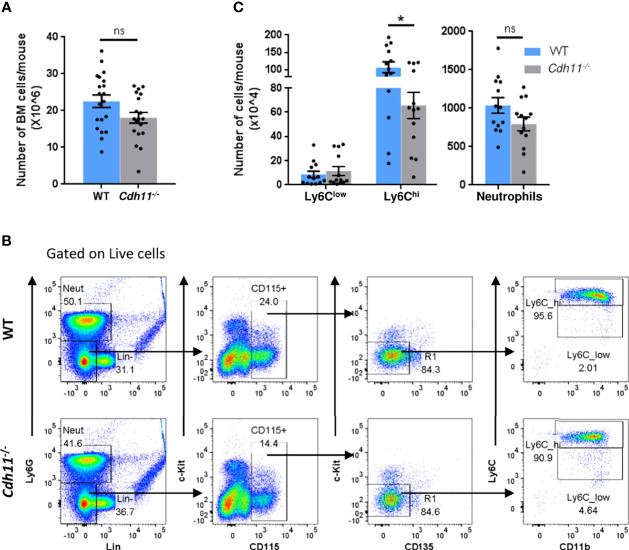
*Cdh11^-/-^* mice have fewer Ly6C^hi^ monocytes in the bone marrow. **(A)** Total number of bone marrow cells from WT (n=10) or *Cdh11^-/-^* (n=10) mice. Data are pooled from three independent experiments. **(B)** Representative flow gating strategy to identify Ly6C^hi^ and Ly6C^low^ monocytes in the bone marrow of WT or *Cdh11^-/-^* mice. **(C)** Total number of Ly6C^hi^ and Ly6C^low^ monocytes, and neutrophils in the bone marrow per mouse. Total numbers of each monocyte subset and neutrophils were calculated by multiplying the frequency of live (determined from flow cytometry) by the total number of lung cells (obtained using a hemocytometer). Data are pooled from four independent experiments and represent mean ± SEM, n=13 mice for each genotype. Statistical significance was assessed using two-tailed unpaired parametric Student’s *t*-test. *P<0.05, ns, non-significant.

The role of CDH11 on myeloid cell development has not yet been investigated. Given that *Cdh11^-/-^* mice have fewer Ly6C^hi^ monocytes in the lungs compared to WT mice before and after bleomycin administration, we hypothesized that CDH11 may play a role in monocyte production. To test this hypothesis, we assessed whether the reduced number of Ly6C^hi^ monocytes in the lungs of naive *Cdh11^-/-^* mice was caused by a reduced production of these cells in the bone marrow. We first assessed the cellularity of the bone marrow and found no significant difference in the total number of bone marrow cells in *Cdh11^-/-^* mice compared to WT control mice (**Figure 3A**). We then analyzed the population of Ly6C^hi^ monocytes in the bone marrow by flow cytometry using the gating strategy outlined in **Figure 3B**. We show that in the bone marrow, the total number of Ly6C^hi^ monocytes (identified as Lin^-^ Ly6G^-^ CD115^+^ c-Kit^-^ CD135^-^ CD11b^+^ Ly6C^hi^) was also significantly reduced in *Cdh11^-/-^* mice compared to WT control mice (**Figure 3C**), suggesting a reduced production of Ly6C^hi^ monocytes in the bone marrow. No difference in the Ly6C^low^ monocytes or neutrophils were observed.

**Figure 4:**
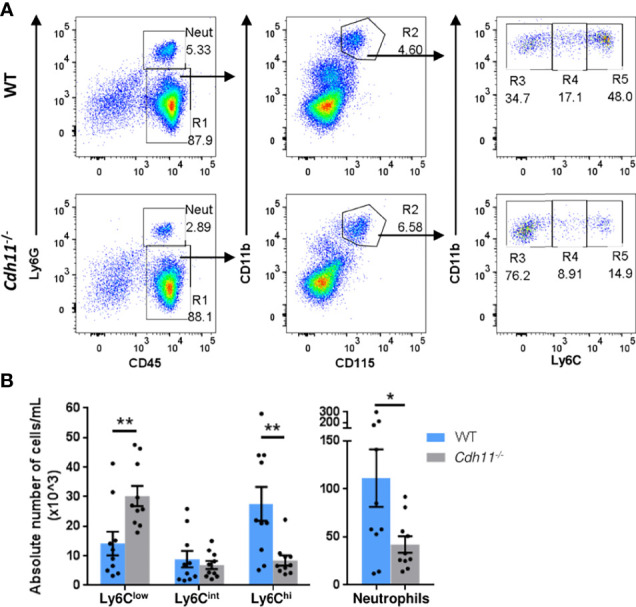
*Cdh11^-/-^* mice have fewer Ly6C^hi^ monocytes in the peripheral blood. **(A)** Representative flow gating strategy to identify Ly6C^hi^, Ly6C^int^, and Ly6C^low^ monocytes, and neutrophils in the peripheral blood of WT or *Cdh11^-/-^* mice. **(B)** Absolute number of each monocyte subset and neutrophils per mL for each mouse. Data are pooled from three independent experiments and represent mean ± SEM, n= 10 mice for each genotype. Statistical significance was assessed using two-tailed unpaired parametric Student’s *t*-test. *P<0.05, **P<0.01. Absolute numbers were calculated using CountBright counting beads.

To further determine the role of CDH11 in monocyte production, we examined the monocyte subsets in the peripheral blood of WT and *Cdh11^-/-^* mice using flow cytometric analysis. *Cdh11^-/-^* mice had significantly fewer numbers of Ly6C^hi^ monocytes in the blood compared to WT control mice (**Figures 4A, B**), consistent with our findings in the bone marrow, and further suggests that CDH11 is required for the development of Ly6C^hi^ monocytes. Interestingly, *Cdh11^-/-^* mice had increased numbers of Ly6C^low^ monocytes and fewer neutrophils in the blood compared to WT mice. No difference in the number of Ly6C^int^ monocytes was observed between the two groups of mice.

**Figure 5:**
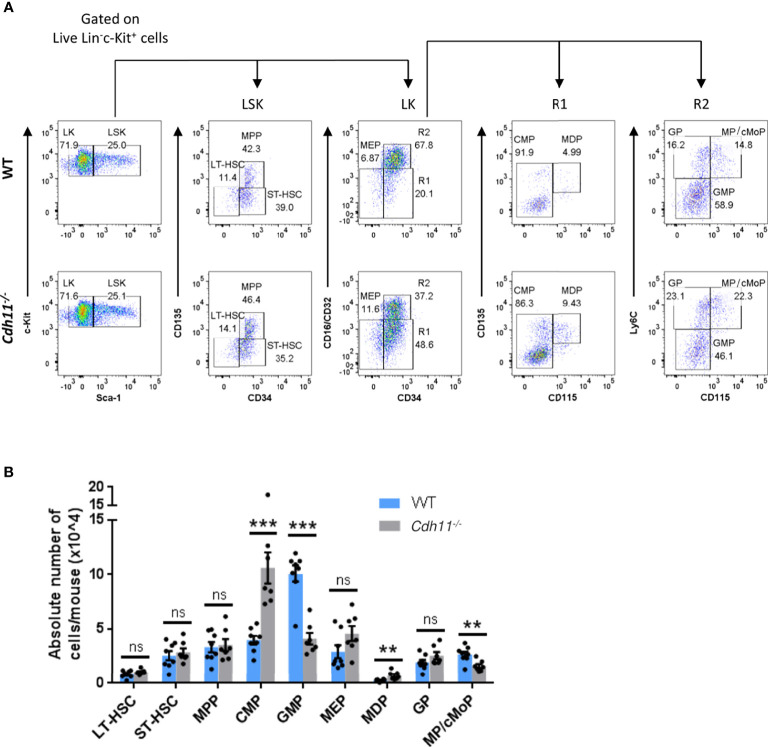
*Cdh11^-/-^* mice have a reduced GMP population and an expanded CMP population in the bone marrow. **(A)** Representative flow cytometry plots showing the gating strategy for identification of LT-HSC, ST-HSC, and MPP cells within the Lin^-^Sca-1^+^c-Kit^+^ (LSK) compartment, and CMP, GMP, MDP, MEP cells and its downstream progenitors, GP and MP/cMoP, within the Lin^-^Sca-1^-^c-Kit^+^ (LK) compartment of bone marrow cells from WT and *Cdh11^-/-^* mice. Gates containing multiple progenitor populations are labeled as R1 and R2. Gates containing a single progenitor are labeled with the included progenitor subset. **(B)** Absolute numbers of each progenitor subset in the bone marrow per mouse. Data are pooled from two independent experiments and represent mean ± SEM of n=8 WT and n=7 *Cdh11^-/-^* mice. Statistical significance was assessed using two-tailed unpaired parametric Student’s *t*-test or the unpaired nonparametric Mann-Whitney test. **P<0.01, ***P<0.001, ns, non-significant. Absolute numbers were calculated using CountBright counting beads.

The reduced numbers of Ly6C^hi^ monocytes in the bone marrow and peripheral blood of *Cdh11^-/-^* mice at steady state suggests that *Cdh11^-/-^* mice may have reduced bone marrow progenitor populations. To investigate this, we quantified the progenitor cell populations in *Cdh11^-/-^* mice compared to WT controls using the surface markers and gating strategy outlined in **Figure 5A**. We found similar numbers of long-term hematopoietic stem cells (LT-HSCs), short-term HSCs (ST-HSCs), multipotent progenitors (MPPs), and megakaryocytic-erythroid progenitors (MEPs) in the bone marrow of WT and *Cdh11^-/-^* mice. However, *Cdh11^-/-^* mice had a significant increase in numbers of common myeloid progenitors (CMPs) and monocyte-DC progenitors (MDPs), and a reduced number of granulocyte-monocyte progenitors (GMPs) relative to the WT control (**Figure 5B**). Further analysis of progenitors downstream of GMPs revealed a significant reduction in a population of cells containing both monocyte-committed progenitors (MPs) and common monocyte progenitors (cMoPs) but no difference in the granulocyte-committed progenitors (GPs). Collectively, these data suggest that loss of CDH11 impairs the differentiation of CMPs towards the myeloid lineage.

### Loss of CDH11 Impairs the Differentiation of Monocytes to Macrophages and Polarization of M2 Macrophages

**Figure 6:**
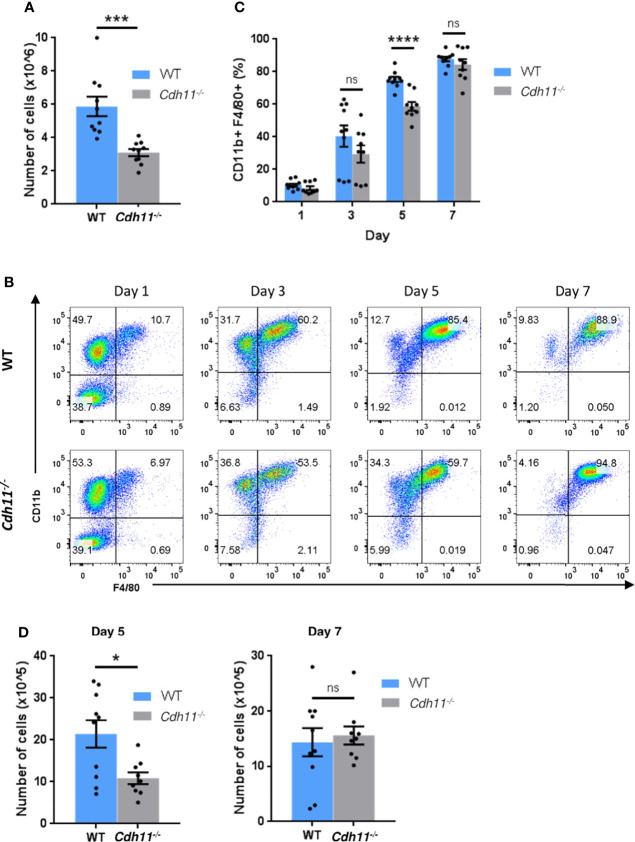
CDH11 deficiency impairs the differentiation of bone marrow cells into macrophages. **(A)** Total number of cells generated from *Cdh11^-/-^* (n=10) or WT (n=10) bone marrow (BM) cells cultured in 50 ng/mL M-CSF for 7 days. **(B)** Representative flow cytometry plots of CD11b^+^ F4/80^+^ macrophage populations generated after culturing *Cdh11^-/-^* (n=9) or WT (n=10) BM cells in 20 ng/mL M-CSF for 1, 3, 5, and 7 days. **(C)** Percentages of CD11b^+^ F4/80^+^ macrophages generated from BM cells at each time point. **(D)** Total number of cells generated from *Cdh11^-/-^* (n=9) or WT (n=10) BM cells cultured in 20 ng/mL M-CSF for 5 and 7 days. Data are pooled from three independent experiments and represent the mean ± SEM of the indicated numbers of mice. Statistical significance was assessed using two-tailed unpaired parametric Student’s *t*-test. *P<0.05, ***P<0.001, ****P<0.0001, ns, non-significant.

Given that monocytes differentiate into macrophages and that *Cdh11^-/-^* mice have fewer Ly6C^hi^ monocytes, IMs, and MoAMs in the lungs after bleomycin administration compared to WT mice, we next investigated the role of CDH11 in macrophage differentiation. We isolated bone marrow cells from *Cdh11^-/-^* and WT mice and differentiated them into macrophages (bone marrow-derived macrophages [BMDM]) using M-CSF. After culturing the bone marrow cells with 50 ng/mL M-CSF for 7 days, we found that bone marrow cells isolated from *Cdh11-/-* mice generated significantly fewer numbers of BMDMs compared to that from WT mice (**Figure 6A**). However, flow analysis showed a similar proportion of predominantly CD11b^+^ F4/80^+^ macrophages (**Supplementary Figure 5**). We then assessed the generation of mature CD11b^+^ F4/80^+^ macrophages at various time points using 20 ng/mL M-CSF and found that *Cdh11^-/-^* bone marrow cells generated fewer cells and a significantly lower proportion of mature CD11b^+^ F4/80^+^ macrophages compared to WT cells after 5 days culture with 20 ng/mL M-CSF (**Figures 6B–D**). However, after 7 days culture in 20 ng/mL M-CSF, similar numbers of mature CD11b^+^ F4/80^+^ macrophages were generated from *Cdh11^-/-^* and WT cells. Given that WT cells cultured for 7 days in 50 ng/mL M-CSF generated significantly more macrophages than those cultured in 20 ng/mL M-CSF (**Figures 6A, D**), it is possible that the lower concentration of M-CSF led to growth arrest and/or cell death in these cultures. While the reduced proportion of mature CD11b^+^ F4/80^+^ macrophages generated in the *Cdh11^-/-^* cultures on day 5 suggests a role for CDH11 in the differentiation of monocyte precursors into macrophages, the reduced number of cells generated in *Cdh11^-/-^* cultures may also be due to reduced proliferation, increased cell death, and/or reduced adherence to plastic. Further studies will need to be performed to assess the contribution of CDH11 in proliferation, apoptosis, and adhesion.

**Figure 7:**
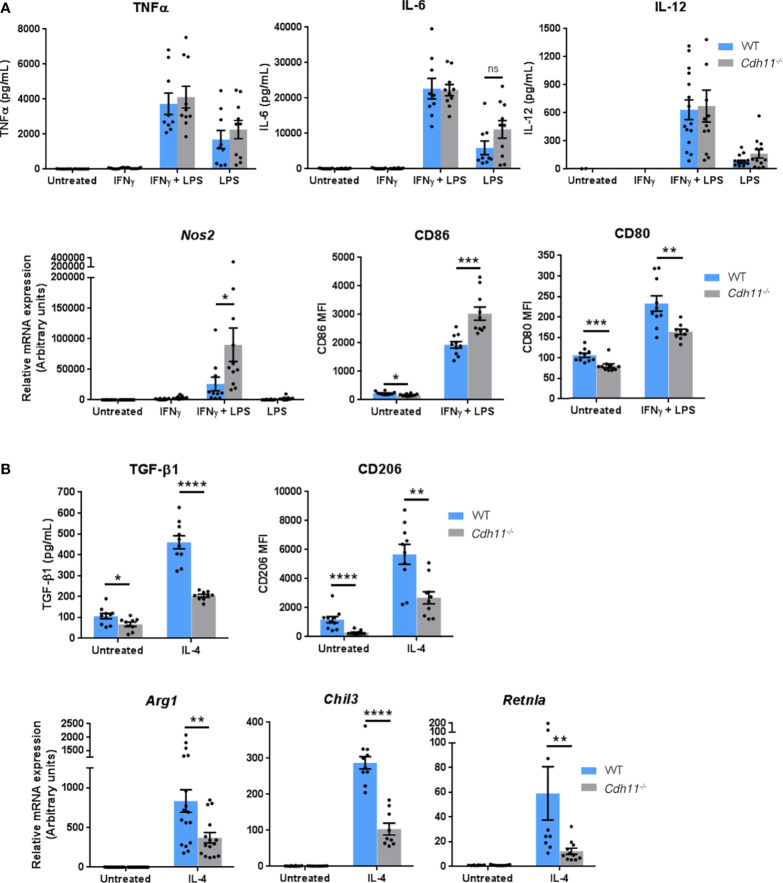
CDH11 deficiency attenuates macrophage M2 polarization *in vitro*. *Cdh11^-/-^* or WT BMDMs (n=9-15) were stimulated with IFNγ and LPS to promote M1 polarization or IL-4 to promote M2 polarization. **(A)** Secretion of M1 pro-inflammatory cytokines TNFα, IL-6, and IL-12 in culture supernatants were measured by ELISA, expression of *Nos2* was measured by real-time PCR, and expression of CD86 and CD80 were assessed by flow cytometry. **(B)** Secretion of M2 anti-inflammatory cytokine TGF-β1 in culture supernatants was measured by ELISA, expression of CD206 was assessed by flow cytometry, and expression of M2 signature genes *Arg1*, *Chil3*, and *Retnla* were measured by real-time PCR. Data are pooled from three to four independent experiments and represent mean ± SEM. Statistical significance was assessed using two-tailed unpaired parametric Student’s *t*-test. *P<0.05, **P<0.01, ***P<0.001, ****P<0.0001, ns; non-significant.

To further investigate the role of CDH11 in macrophage differentiation, we next examined the effects of loss of CDH11 on macrophage polarization. We polarized BMDMs towards the M1 phenotype by incubation with IFNγ and LPS and found no significant difference in the production of the pro-inflammatory cytokines TNFα, IL-6, and IL-12 between *Cdh11^-/-^* BMDMs compared to WT BMDMs (**Figure 7A**). However, the expression of the M1-associated markers *Nos2* and CD86 were significantly increased whereas the expression of CD80 was reduced in *Cdh11^-/-^* BMDMs compared to WT controls. We then polarized BMDMs towards the M2 phenotype by incubation with IL-4 and found decreased production of the anti-inflammatory cytokine TGF-β1 and a significant reduction in expression of the prototypical M2 markers CD206, *Chil3*, *Arg1*, and *Retnla* in *Cdh11^-/-^* BMDMs compared to WT BMDMs (**Figure 7B**). These data suggest that CDH11 enhances the polarization of macrophages towards the M2 phenotype.

**Figure 8:**
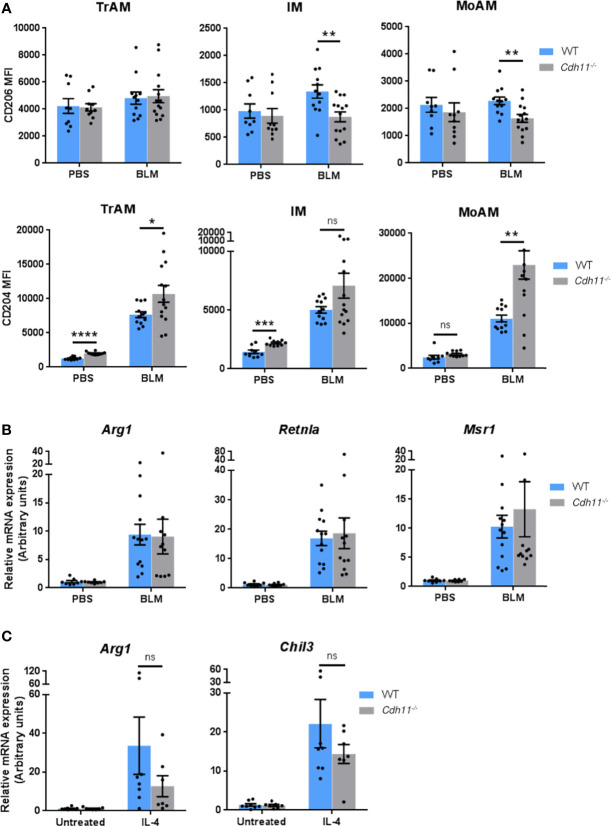
CDH11 deficiency does not affect M2 macrophage polarization *in vivo*. **(A)** CD204 and CD206 expression on TrAMs, IMs, and MoAMs in *Cdh11^-/-^* or WT mouse lungs were analyzed by flow cytometry 21 days after bleomycin (BLM) administration and expressed as mean fluorescent intensity (MFI). n=9 (WT PBS), n=12 (WT BLM), n=10 (*Cdh11^-/-^* PBS), n=14 (*Cdh11^-/-^* BLM). **(B)** Expression of *Arg1*, *Retnla*, and *Msr1* in whole lungs isolated from *Cdh11^-/-^* or WT mice treated with bleomycin were measured by real-time PCR. n=8 (WT PBS), n=13 (WT BLM), n=8 (*Cdh11^-/-^* PBS), n=11 (*Cdh11^-/-^* BLM). Data are pooled from three to four independent experiments. **(C)** Primary alveolar macrophages from *Cdh11^-/-^* (n=7) or WT (n=8) mice were isolated from bronchoalveolar lavage fluid and cultured with IL-4 for 24 hrs. Expression of *Arg1* and *Chil3* were measured by real-time PCR. Data are pooled from two independent experiments. All data are expressed as mean ± SEM. Statistical significance was assessed using two-tailed unpaired parametric Student’s *t*-test. *P<0.05, **P<0.01, ***P<0.001, ****P<0.0001, ns, non-significant.

We then investigated whether CDH11 is required for the polarization of M2 macrophages *in vivo* in the IP bleomycin-induced pulmonary fibrosis model. We used flow cytometry to assess the expression of the M2 associated markers CD206 and CD204 on TrAMs, IMs, and MoAMs. We found high expression of CD206 on all macrophage subsets in WT and *Cdh11^-/-^* mice (**Figure 8A**). However, bleomycin administration did not exert significant impact on CD206 expression in this model. In contrast, CD204 expression was markedly upregulated on all macrophage subsets following bleomycin administration in WT mice and was further upregulated on TrAMs and MoAMs in bleomycin-treated *Cdh11^-/-^* mice. Analysis of classic M2 signature gene expression in whole lung isolated from bleomycin-treated *Cdh11^-/-^* and WT mice showed increased expression of *Arg1*, *Retnla*, and *Msr1* in WT mice treated with bleomycin (**Figure 8B**). However, lungs from *Cdh11^-/-^* mice treated with bleomycin expressed similar levels of *Arg1*, *Retnla*, and *Msr1*. We also isolated alveolar macrophages from bronchoalveolar lavage fluid collected from *Cdh11^-/-^* and WT mice and cultured them with IL-4 to induce M2 differentiation but found no significant difference in the expression of *Arg1* or *Chil3* (**Figure 8C**). Collectively, our *in vitro* data on BMDMs show regulation of M2 macrophage polarization by CDH11. However, our *in vivo* data show that CDH11 may not be regulating the M2 program during pulmonary fibrosis.

### Loss of CDH11 in Macrophages Impairs Phagocytic Function

**Figure 9:**
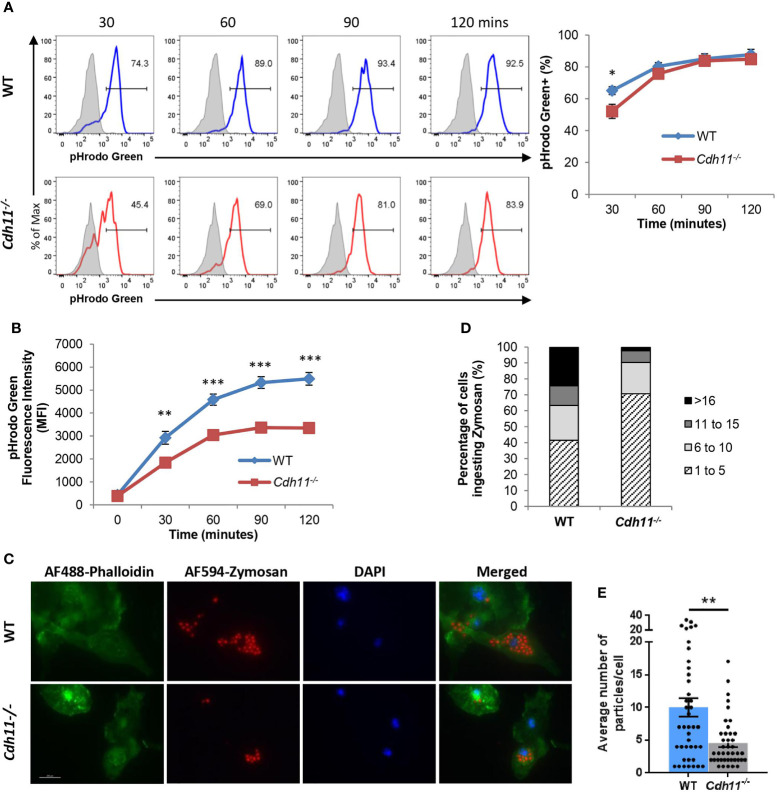
*Cdh11*-deficient macrophages have decreased phagocytic function. **(A)** *Cdh11^-/-^* (n=8) or WT (n=9) BMDMs were incubated with pHrodo-green labeled zymosan bioparticles for the indicated times and phagocytosis was assessed by flow cytometry. Representative histograms and quantification showing percentage of BMDMs containing positive pHrodo-green fluorescence signal. Blue open histogram = WT BMDMs, red open histogram = *Cdh11^-/-^* BMDMs, shaded grey histogram = BMDMs incubated with bioparticles on ice. **(B)** Quantification of the shift in mean fluorescence intensity (MFI). **(C)** Phagocytosis activity in *Cdh11^-/-^* or WT BMDMs was assessed by immunofluorescence using AF594-conjugated zymosan bioparticles (red). F-actin was stained with AF488-conjugated phalloidin (green). Nuclei were stained with DAPI (blue). Representative fluorescence images from three independent experiments are shown. Scale bar is 200 µm. **(D, E)** Quantitative analysis of numbers of particles internalized by *Cdh11^-/-^* or WT BMDMs. Data are expressed as percentages of macrophages ingesting specific numbers of particles **(D)** or average number of particles ingested by each macrophage **(E)**. The number of particles ingested into 41 BMDM cells were counted. Data are pooled from three independent experiments and expressed as mean ± SEM. Statistical significance was assessed using two-tailed unpaired parametric Student’s *t*-test or unpaired non-parametric Mann Whitney U-test. *P<0.05, **P<0.01, ***P<0.001.

Phagocytosis is one of the key functions of macrophages and important for maintenance of normal tissue homeostasis as well as clearance of microbial pathogens during infections (33, 34). M2 macrophages have been shown to have higher phagocytic activity than M1 macrophages (35–39). Given the dampened M2 phenotype of *Cdh11^-/-^* BMDMs, we next assessed the effect of CDH11 on their phagocytic function. We first analyzed phagocytosis using pHrodo-green Zymosan bioparticles by flow cytometry. pHrodo-green bioparticles are non-fluorescent at neutral pH and fluorescence intensity increases as they are taken up into acidic vesicles. WT and *Cdh11^-/-^* BMDMs were incubated with pHrodo-green Zymosan bioparticles for the indicated time points and phagocytosis was assessed by the increase in green fluorescence signal. We found a significantly lower percentage of pHrodo-green positive cells in *Cdh11^-/-^* BMDMs after 30 minutes of incubation compared to WT BMDMs (**Figure 9A**). No significant difference in the fluorescence signal was observed at 60, 90, and 120 minutes of incubation. Analysis of the shift in fluorescence intensity showed significantly decreased mean fluorescent intensity (MFI) in *Cdh11^-/-^* BMDMs compared to WT BMDMs at all time points (**Figure 9B**), suggesting decreased ingestion of the pHrodo-green Zymosan bioparticles by *Cdh11^-/-^* BMDMs. To confirm these observations, we also evaluated phagocytosis of fluorescently labeled bioparticles by microscopy. WT and *Cdh11^-/-^* BMDMs were incubated with Alexa-Fluor-594-conjugated Zymosan bioparticles and the number of bioparticles ingested were counted. Similarly to the flow cytometry data, *Cdh11^-/–^* BMDMs ingested fewer numbers of bioparticles compared to WT BMDMs (**Figures 9C–E**). Alveolar macrophages isolated from *Cdh11^-/-^* mice were also less efficient at phagocytosing the bioparticles compared to those from WT mice (**Supplementary Figure 6**). These data show that *Cdh11^-/-^* macrophages have impaired phagocytic function compared to WT macrophages.

## Discussion

CDH11 expression is upregulated on alveolar macrophages in the fibrotic lung (23, 29) but the role of CDH11 on macrophage biology is unknown. In this study, we use the bleomycin-induced pulmonary fibrosis model to investigate the role of CDH11 on macrophage biology. Using flow cytometric analysis, we show that in the IP bleomycin model of pulmonary fibrosis, *Cdh11^-/-^* mice have reduced numbers of MoAMs and IMs compared to WT mice. Several studies have shown that monocyte-derived macrophages are derived from Ly6C^hi^ monocytes during lung injury (3, 4, 18, 40) with IMs believed to be a transition process between monocytes and alveolar macrophages (5, 41, 42). Therefore, we hypothesized that the reduced numbers of MoAMs and IMs may be due to fewer Ly6C^hi^ monocytes. Accordingly, we found fewer Ly6C^hi^ monocytes in the lungs of bleomycin-treated *Cdh11^-/-^* mice compared to WT mice. Additionally, naive *Cdh11^-/-^* mice also have fewer Ly6C^hi^ monocytes in the bone marrow and peripheral blood. Interestingly, *Cdh11^-/-^* mice have similar numbers of neutrophils in the bone marrow as WT mice, suggesting a specific role for CDH11 in the development of Ly6C^hi^ monocytes.

Ly6C^hi^ monocytes in the bone marrow can arise from the multilineage CMPs *via* two independent pathways: GMPs, which produce neutrophil-like Ly6C^hi^ monocytes, or MDPs, which produce Ly6C^hi^ monocytes that are capable of producing monocyte-derived dendritic cells (43). Both monocyte lineages produce macrophages that may be functionally distinct. We assessed the hematopoietic progenitors in *Cdh11^-/-^* and WT mice and found increased numbers of CMPs and MDPs and fewer numbers of GMPs in *Cdh11^-/-^* mice relative to WT mice. We also found reduced numbers of MPs, produced by GMPs, and cMoPs, produced by MDPs (44), in *Cdh11^-/-^* mice compared to WT mice. Although MPs and cMoPs are not yet able to be distinguished using known surface marker expression, RNA sequencing has revealed that they are distinct cell types and that they produce distinct Ly6C^hi^ monocytes (43). These data suggest that loss of CDH11 impairs the differentiation of CMPs towards the myeloid lineage. *In vitro* functional assays on sorted progenitors are needed to confirm this differentiation defect. When bone marrow cells isolated from *Cdh11^-/-^* and WT mice were cultured in the presence of 20 ng/mL M-CSF, a reduced proportion of mature CD11b^+^ F4/80^+^ macrophages was generated from *Cdh11^-/-^* cells after 5 days culture compared to WT cells, further suggesting that loss of CDH11 impairs monocyte to macrophage differentiation. CDH11 has previously been reported to play a role in cellular proliferation and differentiation of mesenchymal cells (45–49). Therefore, the fewer number of mature macrophages generated from *Cdh11^-/-^* bone marrow can also be attributed to decreased proliferation.

We have previously shown that alveolar macrophages isolated from *Cdh11^-/-^* mice produce significantly less TGF-β compared to those from WT mice (23). Bronchoalveolar lavage fluids collected from *Cdh11^-/-^* mice challenged with bleomycin or CDH11 antibody-treated mice given bleomycin also had significantly less TGF-β compared to those collected from WT mice given bleomycin (23). Given that M2 activated macrophages are a major cellular source of TGF-β production and CDH11 expression has been reported on M2 activated macrophages (27, 29), we hypothesized that CDH11 may be regulating TGF-β production through the M2 program. Analysis of BMDMs from *Cdh11^-/-^* mice stimulated with IL-4 showed a significant reduction in the expression of all M2-associated markers compared to those from WT mice. Although the concept of M2 polarization is an oversimplification of the complex gene expression changes that occurs *in vivo* during lung fibrosis (5, 50), we show increased expression of *Arg1*, *Retnla*, and *Msr1* (CD204) in lungs from WT mice given bleomycin, suggesting a contribution of an M2 phenotype in the development of pulmonary fibrosis. We also detected increased expression of *Arg1* and *Chil3* on IL-4 stimulated alveolar macrophages isolated from WT mice. However, no significant difference in expression of these classic M2 signature genes were detected in lungs from *Cdh11^-/-^* mice treated with bleomycin or IL-4 stimulated *Cdh11^-/-^* alveolar macrophages compared to those from WT mice. While our *in vitro* data suggests that CDH11 may regulate the M2 phenotype of macrophages, our *in vivo* data argues against an exclusive role for regulation of M2 polarization by CDH11 in the pathogenesis of pulmonary fibrosis and suggests that regulation of monocyte-derived macrophage development by CDH11 is more likely a component of the mechanism of lung fibrosis.

During lung injury, apoptotic inflammatory cells are removed by recruited alveolar macrophages (51, 52). Uptake of apoptotic cells by macrophages also induces an M2 phenotype and secretion of TGF-β1 (53–55). Given that M2 macrophages have higher phagocytic activity than M1 macrophages (35–39), the expression of CDH11 on M2 macrophages (27, 29), and a role for CDH11 on M2 macrophage polarization *in vitro*, we also investigated the role of CDH11 on the phagocytic function of macrophages. We found that loss of CDH11 significantly reduced phagocytic capability, providing further evidence that *Cdh11*-deficient macrophages may have a reduced M2 phenotype. Further studies are needed to determine whether regulation of TGF-β1 production by CDH11 is mediated through ingestion of apoptotic cells.

In conclusion, our study demonstrates that CDH11 regulates monocyte-derived macrophage development, polarization to a profibrotic M2 phenotype, and phagocytic function. Our data suggest that CDH11 deficiency impairs the differentiation of CMPs towards the myeloid lineage which results in the production of fewer Ly6C^hi^ monocytes and may lead to the recruitment of fewer monocyte-derived macrophages and attenuation of pulmonary fibrosis in *Cdh11^-/-^* mice. Our *in vitro* BMDM differentiation and phagocytosis studies also suggest a reduced M2 phenotype in *Cdh11^-/-^* macrophages which may be responsible for the decreased production of TGF-β1 in *Cdh11^-/-^* mice. Collectively, our study provides insight into the role of CDH11 in macrophages and pulmonary fibrosis.

## Data Availability Statement

The original contributions presented in the study are included in the article/**Supplementary Material**. Further inquiries can be directed to the corresponding author.

## Ethics Statement

The animal study was reviewed and approved by Institutional Animal Care and Use Committee (IACUC) of Baylor College of Medicine.

## Author Contributions

ST and SA were responsible for the conception and design of the study. ST performed the experiments with help from TC, MP, and JS. ST analyzed the data, interpreted the results, and wrote the manuscript. All authors contributed to the article and approved the submitted version.

## Funding

This project was supported by the National Institute of Health (NIH) grant R01AR062056-01A1 (to SA), the Scleroderma Foundation Established Investigator Award (to SA), and the Cytometry and Cell Sorting Core at Baylor College of Medicine with funding from the CPRIT Core Facility Support Award (CPRIT-RP180672), the NIH (CA125123 and RR024574) and the assistance of Joel M. Sederstrom. The Krist Foundation provided financial support for experiments conducted in this manuscript.

## Conflict of Interest

The authors declare that the research was conducted in the absence of any commercial or financial relationships that could be construed as a potential conflict of interest.

## Publisher’s Note

All claims expressed in this article are solely those of the authors and do not necessarily represent those of their affiliated organizations, or those of the publisher, the editors and the reviewers. Any product that may be evaluated in this article, or claim that may be made by its manufacturer, is not guaranteed or endorsed by the publisher.
