# Supplementary material for: Cadherin-11 Regulates Macrophage Development and Function
Source: Front Immunol. 2022 Feb 8;13:795337. doi: 10.3389/fimmu.2022.795337 (PMC8860974; doi:10.3389/fimmu.2022.795337)
Supplement: Supplementary file 1 [file DataSheet_1.pdf]

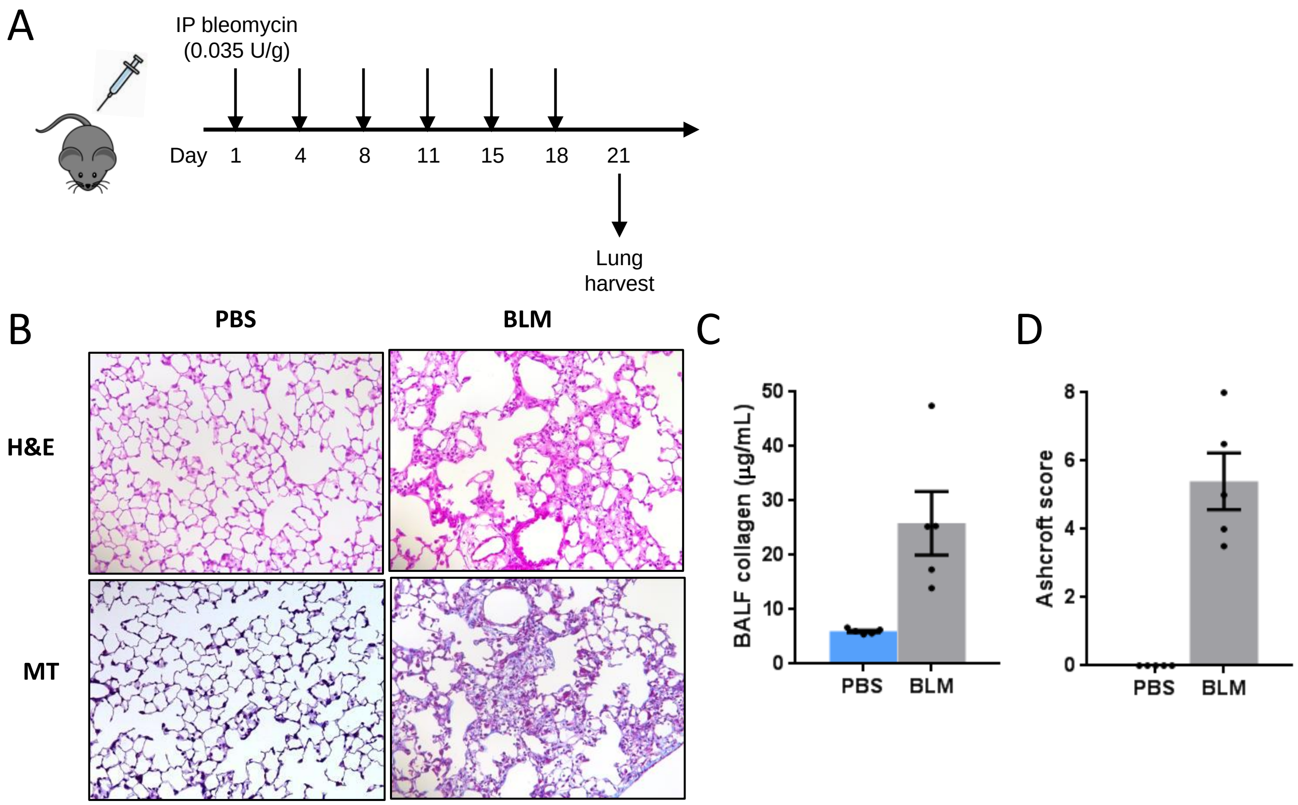

Supplementary Figure 1. Lung fibrosis of wild type mice 21 days after IP bleomycin administration

(A) Schematic of the bleomycin (BLM) dosing strategy. (B) Histological analysis of lung sections from wild type mice 21 days after IP bleomycin administration. Mice treated with PBS were used as control. Representative images of hematoxylin and eosin (H&E) and Masson's Trichrome (MT) staining. (C) Bronchoalveolar lavage fluid (BALF) was collected and soluble collagen content was measured using the Sircol Collagen assay. (D) Lung sections were scored using the modified Ashcroft method to show the overall severity of fibrosis. Data are expressed as mean ± SEM of n=5 mice per group.
