# Supplementary material for: Cadherin-11 Regulates Macrophage Development and Function
Source: Front Immunol. 2022 Feb 8;13:795337. doi: 10.3389/fimmu.2022.795337 (PMC8860974; doi:10.3389/fimmu.2022.795337)
Supplement: Supplementary file 2 [file DataSheet_2.pdf]

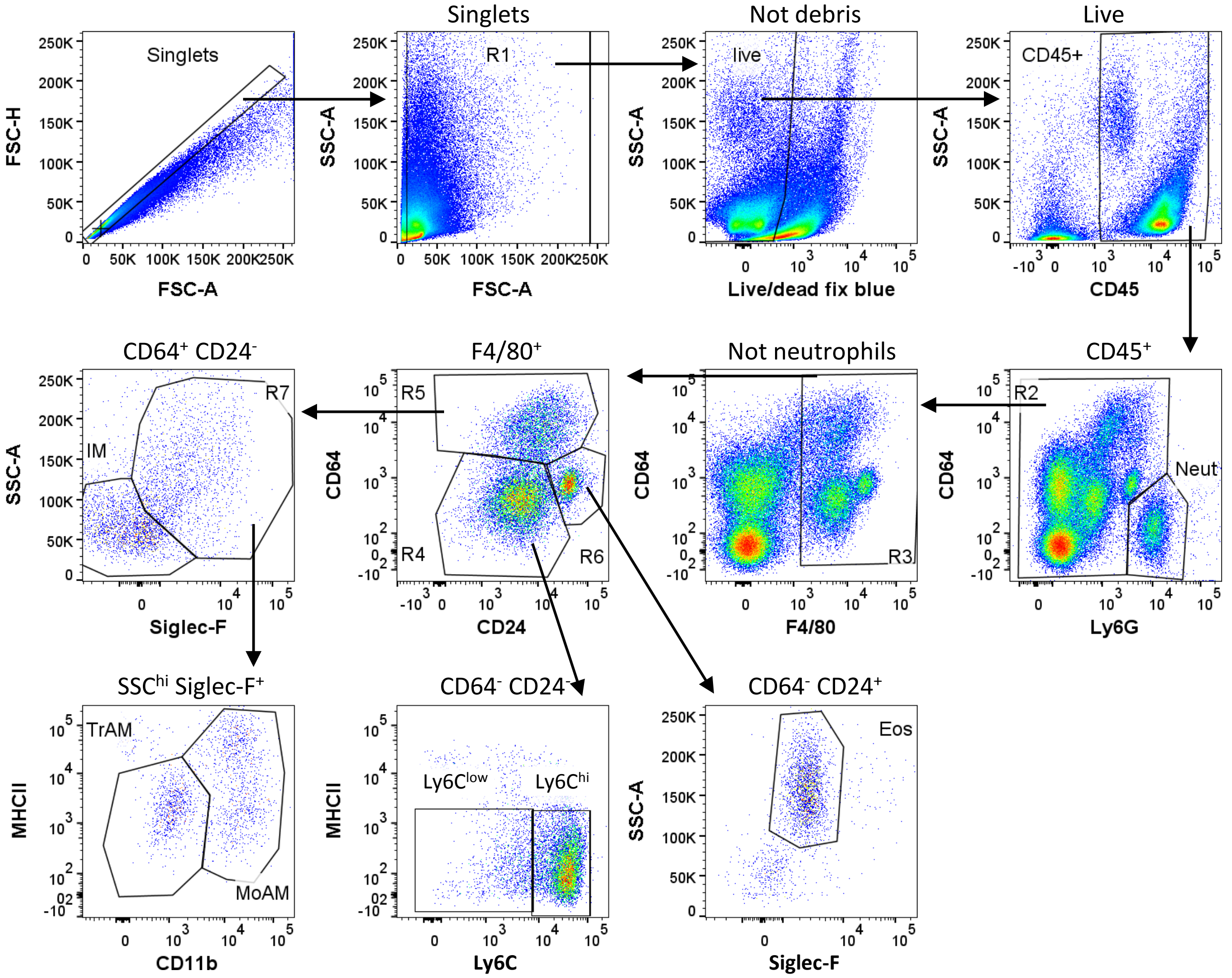

Supplementary Figure 2. Gating strategy for the identification of myeloid cell populations in the mouse lung

Representative gating is shown on a whole lung digest of a wild type mouse 21 days after IP bleomycin administration. After exclusion of doublets and debris, live cells were selected and immune cells were identified by CD45 staining. Tissue-resident alveolar macrophages (TrAM) were identified as Ly6G$^-$ F4/80$^+$ CD64$^+$ CD24$^-$ Siglec-F$^+$ SSC^{hi} CD11b$^-$ whereas monocyte-derived alveolar macrophages (MoAM) are Ly6G$^-$ F4/80$^+$ CD64$^+$ CD24$^-$ Siglec-F$^+$ SSC^{hi} CD11b$^+$. Interstitial macrophages (IM) were identified as Ly6G$^-$ F4/80$^+$ CD64$^+$ CD24$^-$ Siglec-F$^-$ SSC^{low}. Ly6C^{low} and Ly6C^{hi} monocytes are Ly6G$^-$ F4/80$^+$ CD64$^-$ CD24$^-$ MHCII$^-$. Gates containing multiple cell populations are labeled as R1-R7. Gates containing a single cell population are labeled with the included cell type. Neutrophils (Neut), eosinophils (Eos).
