# Supplementary material for: Cadherin-11 Regulates Macrophage Development and Function
Source: Front Immunol. 2022 Feb 8;13:795337. doi: 10.3389/fimmu.2022.795337 (PMC8860974; doi:10.3389/fimmu.2022.795337)
Supplement: Supplementary file 3 [file DataSheet_3.pdf]

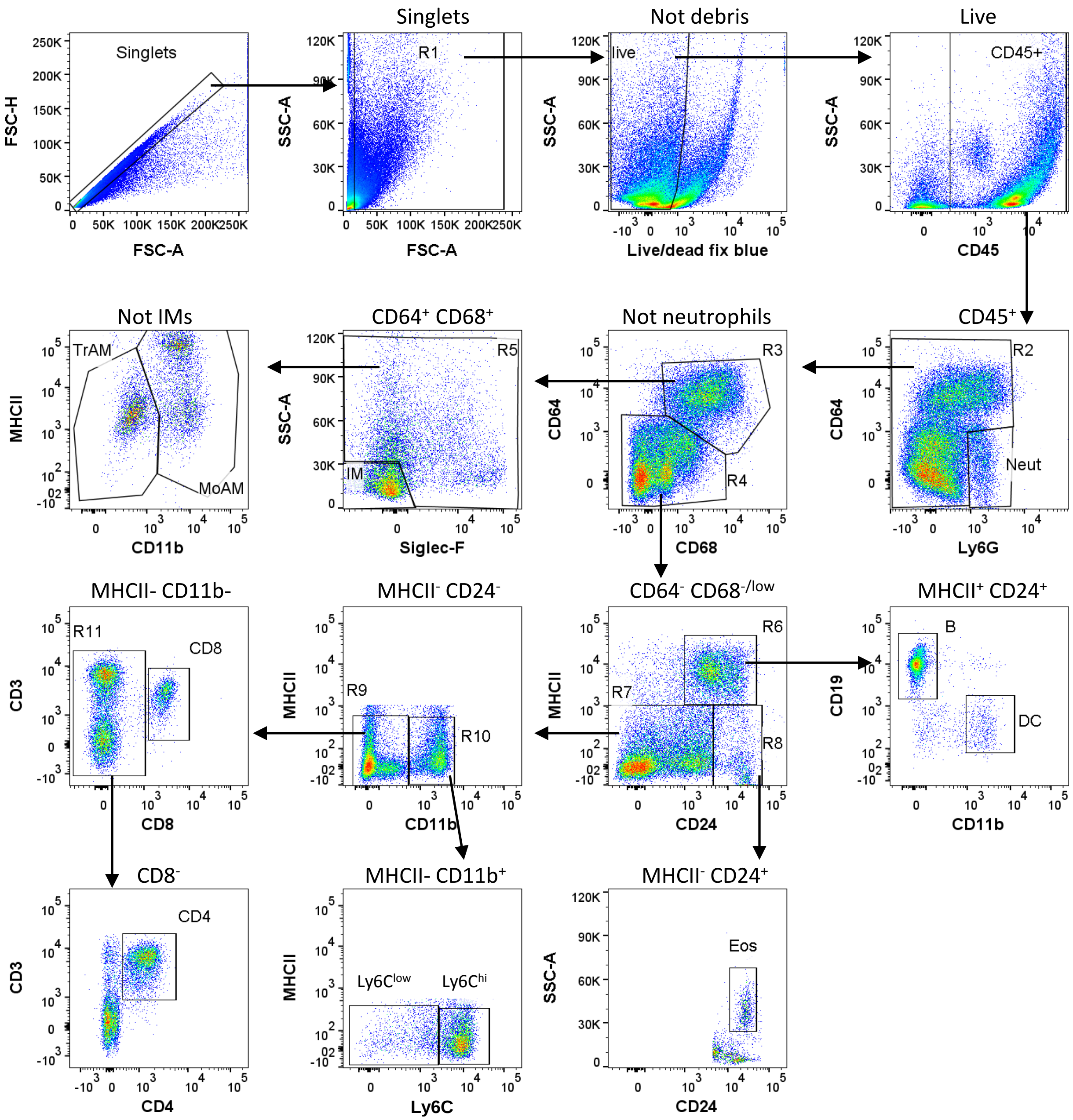

Supplementary Figure 3. Gating strategy for the identification of leukocyte subsets in the mouse lung

Representative gating is shown on a whole lung digest of a wild type mouse 21 days after IP bleomycin administration. Gates containing multiple cell populations are labeled R1-R10. Gates containing a single cell population are labeled with the included cell type. These include neutrophils (Neut), interstitial macrophages (IM), tissue-resident alveolar macrophages (TrAM), monocyte-derived alveolar macrophages (MoAM), dendritic cells (DC), B cells (B), $CD8^{+}$ T cells (CD8), $CD4^{+}$ T cells (CD4), $Ly6C^{low}$ monocytes ($Ly6C^{low}$), $Ly6C^{hi}$ monocytes ($Ly6C^{hi}$), and eosinophils (Eos).
