# Supplementary material for: Cadherin-11 Regulates Macrophage Development and Function
Source: Front Immunol. 2022 Feb 8;13:795337. doi: 10.3389/fimmu.2022.795337 (PMC8860974; doi:10.3389/fimmu.2022.795337)
Supplement: Supplementary file 4 [file DataSheet_4.pdf]

A

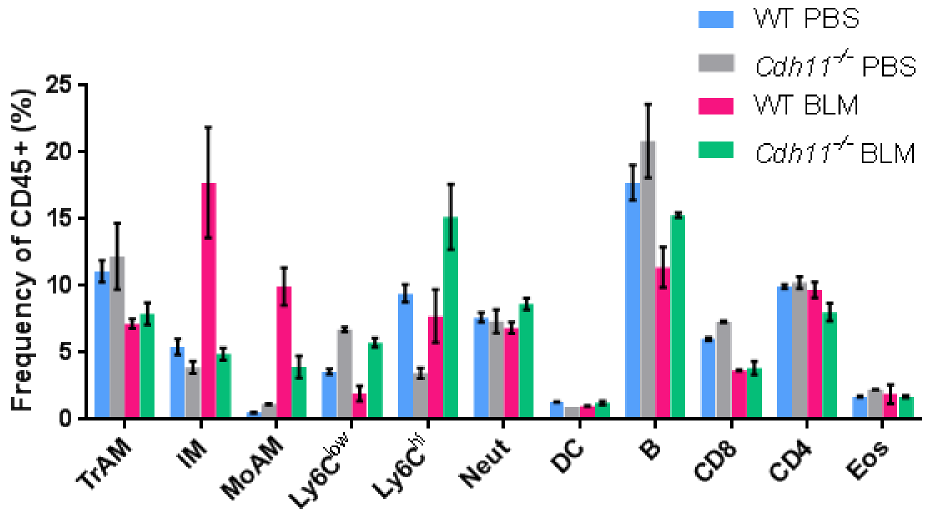

B

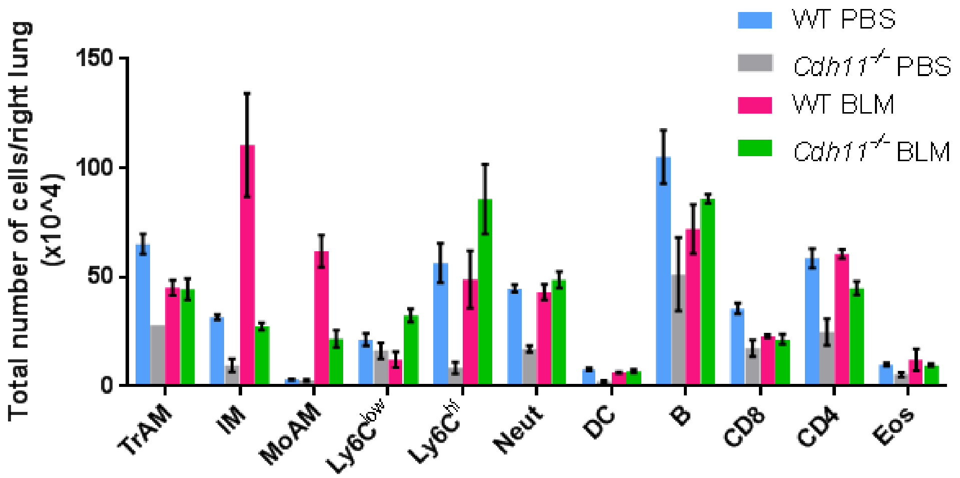

Supplementary Figure 4. Proportions of immune cell populations in the lungs of *Cdh11*$^{-/-}$ and WT mice following 21 days IP bleomycin (BLM) administration.

(A) Frequency of each cell population expressed as percentage of total $CD45^+$ cells. (B) Total number of each cell population per right lung. Data are expressed as mean ± SEM of n=3 mice per group (except *Cdh11*$^{-/-}$ PBS where n=2 mice). Total numbers of each immune cell population were calculated by multiplying the frequency of live (determined from flow cytometry) by the total number of lung cells (obtained using a hemocytometer).
