# Supplementary material for: Cadherin-11 Regulates Macrophage Development and Function
Source: Front Immunol. 2022 Feb 8;13:795337. doi: 10.3389/fimmu.2022.795337 (PMC8860974; doi:10.3389/fimmu.2022.795337)
Supplement: Supplementary file 5 [file DataSheet_5.pdf]

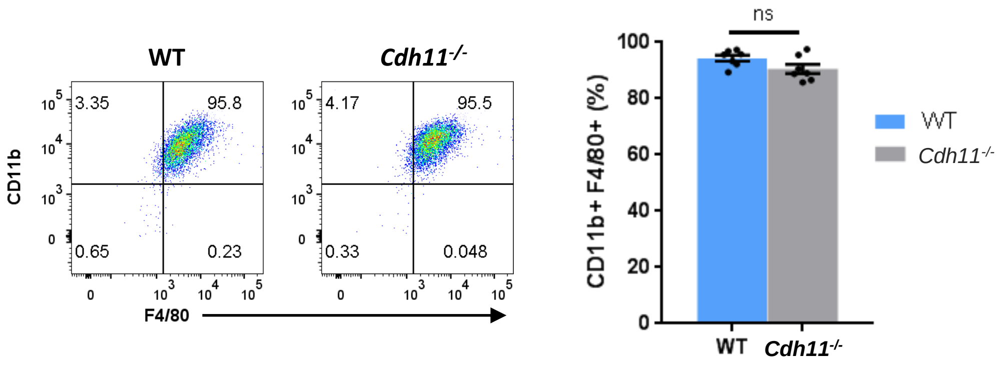

Supplementary Figure 5. CD11b and F4/80 expression on *Cdh11*$^{-/-}$ and WT BMDMs generated with 50 ng/mL M-CSF.

Bone marrow cells from *Cdh11*$^{-/-}$ or WT mice were cultured in 50 ng/mL M-CSF for 7 days. (A) Representative flow cytometry plots of CD11b^{+} F4/80^{+} macrophages. (B) Percentages of CD11b^{+} F4/80^{+} macrophages. Data represent the mean ± SEM , n=7 mice for each genotype. Statistical significance was assessed using two-tailed unpaired parametric Student’s *t*-test. ns; non-significant.
