# Supplementary material for: Cadherin-11 Regulates Macrophage Development and Function
Source: Front Immunol. 2022 Feb 8;13:795337. doi: 10.3389/fimmu.2022.795337 (PMC8860974; doi:10.3389/fimmu.2022.795337)
Supplement: Supplementary file 6 [file DataSheet_6.pdf]

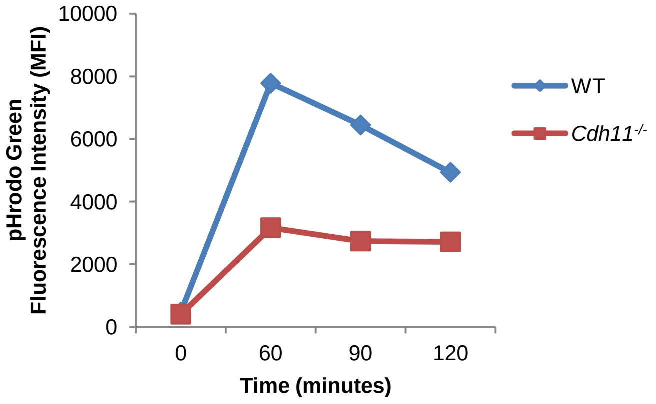

Supplementary Figure 6. ***Cdh11*-deficient alveolar macrophages have decreased phagocytic function**

Alveolar macrophages isolated from BAL fluid collected from *Cdh11*$^{-/-}$ or WT mice (n=5 for each genotype) were pooled and incubated with pHrodo-green zymosan bioparticles. Internalization of zymosan particles were determined by an increase in pHrodo-green mean fluorescence intensity (MFI) at various time points.
